# Influence of Electronic Cigarettes on Selected Physicochemical Properties of Saliva

**DOI:** 10.3390/ijerph19063314

**Published:** 2022-03-11

**Authors:** Dominika Cichońska, Aida Kusiak, Barbara Kochańska, Jolanta Ochocińska, Dariusz Świetlik

**Affiliations:** 1 Department of Periodontology and Oral Mucosa Diseases, Medical University of Gdańsk, Orzeszkowej 18 St., 80-208 Gdańsk, Poland; akusiak@gumed.edu.pl; 2 Department of Conservative Dentistry, Medical University of Gdańsk, Orzeszkowej 18 St., 80-208 Gdańsk, Poland; barbara.kochanska@gumed.edu.pl (B.K.); jolanta.ochocinska@gumed.edu.pl (J.O.); 3 Division of Biostatistics and Neural Networks, Medical University of Gdańsk, Dębinki 1 St., 80-211 Gdańsk, Poland; dariusz.swietlik@gumed.edu.pl

**Keywords:** e-cigarettes, saliva, oral health

## Abstract

(1) Background: Electronic cigarettes are gaining more popularity not only among cigarettes smokers. Firstly, e-cigarettes were perceived as less harmful than traditional cigarettes, however, nowadays, they are arousing more controversy. The aim of this study was to assess the impact of e-cigarette usage on selected physicochemical properties of saliva. (2) Methods: The study population included 128 patients: 40 patients using e-cigarettes, 39 patients smoking traditional cigarettes, and 49 non-smoking patients (non-smokers). Laboratory tests involved verification of saliva values of pH and concentration of total protein, calcium, and phosphates. (3) Results: Among e-cigarette users, the value of pH was lower and the concentration of total protein, calcium, and phosphates was higher than in the group of non-smokers. Statistically significant differences were observed in relation to calcium. Among traditional cigarette smokers, the value of pH was lower, concentrations of total protein and phosphates were higher than in the group of non-smokers. Statistically significant differences were observed in relation to total protein. (4) Conclusions: Saliva of e-cigarette users presents changes in physicochemical composition in comparison to traditional cigarette smokers and non-smokers, however, statistically significant differences were observed only in calcium concentration. Further longitudinal studies on a larger study group should be conducted to assess the effect of observed changes in oral health.

## 1. Introduction

The usage of electronic cigarettes (e-cigarettes) is still a new phenomenon that is constantly increasing around the world. Due to the wide variety of tastes, the possibility to choose and regulate the nicotine content and wide availability, they are encouraging to users, regardless of their age and social origin [1,2,3,4]. According to recent research, cigarette smokers tend to choose e-cigarettes over other nicotine substitutes in smoking cessation. E-cigarette usage is also observed among young adults and adolescents who have never smoked traditional cigarettes before [2,3,4].

E-cigarette is an electronic device providing users with an experience similar to smoking traditional cigarettes that consists of a battery, a vaporization chamber, and a solvent mixture cartridge [5]. The cartridge is a replaceable container to which special liquids are added. The atomizer is the main element of the device, where the liquid is heated and transformed into an aerosol, resembling the visual effect of cigarette smoke [1,6]. Currently, two types of e-cigarettes are available to users: Open-system and closed-system. Open-system e-cigarettes can be refilled with e-liquids available in a considerable variety of flavors and nicotine concentrations. They are usually larger in size than traditional cigarettes and must be charged. Closed-system e-cigarettes cannot be refilled with e-liquids and are usually disposable after usage, however, some of them might also be rechargeable. They are available in a limited variety of nicotine concentrations and flavors. They are also smaller in size than open-system e-cigarettes and bear more resemblance to traditional cigarettes [7]. E-cigarettes were initially perceived as a less harmful alternative to traditional cigarette smoking, however, nowadays they are arousing much controversy and are becoming a subject of scientific research [8]. The main advantage of e-cigarettes is the elimination of carcinogenic components contained in tobacco smoke, such as polycyclic aromatic hydrocarbons, carbon monoxide, and others [9]. The inhalation solution of e-cigarettes consists of propylene glycol and additives, such as glycerol, nicotine, and flavorings [10,11,12]. Both propylene glycol and glycerol are substances approved for use in the food, pharmaceutical, and cosmetic industries [1,13,14], however, heated to high temperatures may produce formaldehyde, which is a highly irritating and carcinogenic component also present in tobacco smoke [6,9,15]. Flavoring substances added to liquids may also pose a threat to e-cigarette users. Fruit, dessert, imitating tobacco, and menthol flavors are added to make e-cigarettes more attractive. Although those flavoring compounds are approved for usage in the food and cosmetics industries, the short- and long-term effects of their inhalation have not been examined yet [1,6,16,17,18,19].

E-cigarette usage might also cause side effects, such as cough, dry throat, conjunctival irritation, and increased heart rate. An adverse effect on the respiratory system has also been observed in the form of an immediate increase in respiratory resistance [1,6].

E-cigarettes can also cause adverse effects in the oral cavity [20,21,22,23,24,25,26,27,28,29,30,31]. Periodontal tissues, such as gingival fibroblasts and periodontal ligaments, are directly exposed to e-cigarette vapor [20,21,22,23]. The in vitro and in vivo research confirmed the cytotoxic effect of e-cigarette aerosol on the epithelial tissue of the oral cavity [23,24,25,26,27], the induction of oxidative stress [28], and an increase in the release of pro-inflammatory cytokines—PGE2, COX2, and IL-8 [20]. E-cigarette usage affects saliva, including saliva’s antibacterial [29] and antioxidant properties [30]. E-cigarette usage also have an impact on oral bacteria, which are essential for the maintenance of homeostasis in the oral cavity [31].

The aim of the study was to assess the impact of e-cigarette usage on selected physicochemical properties of saliva.

## 2. Materials and Methods

### 2.1. Patients’ Population

One hundred twenty-eight patients participated in this study: 40 patients using e-cigarettes (e-cigarettes users), 39 patients smoking traditional cigarettes, and 49 non-smoking patients (non-smokers). All patients who participated in this study were generally healthy people of age 20 to 30. They were patients of the Department of Periodontology and Oral Mucosa Diseases and students at the Medical University of Gdansk, who volunteered for periodontal examination and saliva collection. This research excluded patients with active dental caries, using prosthesis or orthodontic appliances, with periodontal and oral mucosa diseases and diseases that might interfere with the condition of oral mucosa and saliva composition, like diabetes, salivary secretion disorders, and taking medications permanently. Patients treated with antibiotics or steroid medications in the last 6 months and patients consuming alcoholic beverages, tea, or coffee were excluded from the research. E-cigarette users were using open-system e-cigarettes multiple times every day for at least 6 months with small nicotine concentration liquids. Traditional cigarette smokers were smoking at least 10 cigarettes per day for at least 6 months. People smoking both traditional and electronic cigarettes were excluded from the research. The study was conducted in 2018–2019. The study protocol has been approved by the Ethics Committee of the Medical University of Gdansk, Poland (NKBBN/161-386/2017). Ethical aspects of the research followed the World Medical Association Declaration of Helsinki.

### 2.2. Saliva Collection

Unstimulated saliva was collected into a sterile silicon Corning-type test tube from all patients who participated in this study. Unstimulated salivary samples were obtained by expectoration in the absence of chewing movements. Saliva was collected 2 h after food intake [32]. The samples were clarified by centrifugation (2000× *g*; 10 min) and immediately stored for the subsequent determination of pH, total protein, calcium, and phosphates.

### 2.3. Analysis of Saliva

The unstimulated saliva was analyzed in the biochemical laboratory of the Conservative Dentistry Medical University of Gdansk, Poland.

The concentration of hydrogen ions was assessed by using a Fisher pH meter.

Quantification of total protein in saliva samples was done by the Lowry method [33]. In this method, the sensitive reaction of peptide bonds and tyrosine with the Folin–Ciocalteu reagent was used. As a result of the reaction, colored products are formed, and their absorbance is read at a wavelength 750 nm. Protein determination using this method was performed with samples of centrifuged saliva (14,000× *g*, 10 min), 0.1% BSA, Folina–Ciocalteau reagent, CTC reagent (consisting of 0.1% CuSO_4_, 0.2% sodium-potassium tartrate, and 10% Na_2_SO_4_), Reagent A (consisting of 5% SDS, 0.8N NaOH and CTC reagent in a ratio of 2:1:1). Protein concentration in tested samples was calculated from the calibration curve.

Determination of ionized calcium concentration in saliva was done by the ARSENAZO III method [34]. This method uses the metallochromogen Arsenazo III, which binds calcium ions to form a colored complex. The absorbance of color products is measured at a wavelength 650 nm. Arsenazo III is characterized by a strong affinity for calcium ions and does not interfere with other cations which are present in saliva. The color intensity measured at a wavelength 650 nm is proportional to the concentration of calcium in the test sample. Arsenazo III reagent consists of Arsenazo III (0.2 mmol/L), imidazole buffer (100 mmol/L), pH 6.75—Alpha Diagnostics. The reagent is ready for immediate use. Standard is calcium (2.5 mmol/L)—Alpha Diagnostics. Non-centrifuged whole saliva was used for the tests. The reagent was added to the saliva sample in a ratio of 1:100, incubated for 1 min, and then, the absorbance was measured at a wavelength 650 nm. Calcium concentration was calculated from the standard curve.

Determination of inorganic phosphates concentration in saliva was done by the modified Daly and Ertingshausen method [35]. This method ignores the reduction of the phosphomolybdate. The level of formed phosphomolybdate is measured at a wavelength 340 nm and is directly proportional to the concentration of inorganic phosphates. Reagent composition includes ammonium molybdate (0.8 mmol/L), sulfuric acid (430 mmol/L), sodium chloride (77 mmol/L), pH < 1, standard is phosphorus (1.61 mmol/L). Non-centrifuged whole saliva was used for the tests. The reagent was added to the saliva sample in a ratio of 1:100, incubated for 5 min, and then, the absorbance was measured at wavelength 340 nm. The phosphorus concentration was calculated from the standard curve.

### 2.4. Statistical Analysis

The statistical analysis was performed using the statistical suite StatSoft. Inc. (Tulsa, OK, USA) (2014), STATISTICA (data analysis software system) version 12.0. from www.statsoft.com, accessed on 9 May 2014 (2014) and Excel. The significance of the difference between more than two groups was assessed with the Kruskal–Wallis test. In the case of statistically significant differences between two groups, post hoc test Dunn for Kruskal–Wallis was utilized. In all the calculations, the statistical significance values *p* < 0.01 and *p* < 0.05 were used.

## 3. Results

The results of the conducted research are presented in Table 1.

The pH value in e-cigarette users was 7.1 ± 0.7 (range 5.5–8.0, Me = 7.0), in the group of cigarettes smokers, it was 7.2 ± 0.6, (range 5.0–8.0, Me = 7.0), and in the non-smokers group, the result was 7.3 ± 0.8 (range 6.0–8.5, Me = 7.5). The values of pH among all groups were similar, although the median values of pH among e-cigarettes users and traditional cigarette smokers were the same and lower than among non-smokers. Therefore, no statistically significant differences were observed.

The total protein concentration in saliva of e-cigarettes users was 2.3 ± 1.2 (range 0.9–7.5, Me = 1.8), in the group of traditional cigarettes smokers, it was 2.2 ± 0.8 (range 1.1–4.3, Me = 2.1), and among non-smokers, it was 1.7 ± 0.5 (range 1.0–3.0, Me = 1.7). The concentration of total protein in saliva among e-cigarette users and traditional cigarette smokers was higher than in non-smokers. Statistically significant differences on value *p* < 0.05 were observed between traditional cigarette smokers and non-smokers. 

The calcium concentration in saliva of e-cigarettes users was 0.8 ± 0.5 (range 0.2–3.0, Me = 0.8), among traditional cigarettes smokers, it was 0.6 ± 0.3 (range 0.2–1.3, Me = 0.6), and in the group of non-smokers, it was 0.6 ± 0.3 (range 0.1–1.6, Me = 0.6). The concentration of calcium in the group of e-cigarette users was higher than among traditional cigarette smokers and non-smokers. Statistically significant differences on value *p* < 0.01 between e-cigarette users and non-smokers were observed. 

The phosphate concentration in saliva of e-cigarette users was 4.3 ± 2.0 (range 1.1–10.3, Me = 4.1), in the group of traditional cigarettes smokers, it was 4.1 ± 2.3 (range 1.8–10.5, Me = 3.0), and among non-smokers, it was 3.4 ± 1.5 (range 1.3–7.6, Me = 2.9). The concentration of phosphates in groups of e-cigarette users and traditional cigarette smokers was higher than among non-smokers, however, no statistically significant differences were observed. 

## 4. Discussion

Saliva is a secretion that provides homeostasis in the oral environment and is constantly produced by salivary glands [36]. Saliva in 99% consists of water, however, its physicochemical properties are determined by the presence of inorganic and organic substances [37]. The organic components of saliva include proteins, non-protein nitrogenous substances, carbohydrates, lipids, and hormones. Inorganic substances are present in ionic form and include sodium, potassium, calcium, magnesium, chlorine, fluorine, iodine, bicarbonates, and phosphates [36,37]. Saliva’s buffering capacity, which enables acid neutralization and maintenance of adequate pH value, depends on the presence of bicarbonate and phosphate ions. Saliva also consists of glycoproteins, which play a significant role in the protection against mechanical damage by moisturizing the oral mucosa. Due to the presence of antioxidants, such as uric acid, glutathione, catalase, peroxidase, glutathione peroxidase, and superoxide dismutase, saliva is also characterized by the ability to neutralize free oxygen radicals [38,39,40,41]. Saliva also presents antibacterial properties related to the presence of immunoglobulin A, lysozyme, lactoferrin, histamine, and leukocytes [37,42]. The physicochemical properties of saliva can be affected by many factors, such as genetic disorders, including Turner syndrome [43] or diabetes and smoking [44]. It has been proven that chemical compounds found in tobacco smoke or e-cigarette vapor dissolve in saliva and pose an impact on its biochemical composition and function [29,30,45].

The proper saliva pH ranges from 6.2 to 7.6. The maintenance of oral environment pH is related to both the buffering activity of saliva and the constant salivary flow, which enables elimination of acids produced by oral bacteria or delivered with food and beverages [46]. In research conducted by Baliga et al., saliva pH among patients with periodontitis was lower than in the group of patients with no periodontal disorders, which led to a conclusion that saliva pH is related to periodontal diseases and might be perceived as a diagnostic biomarker of periodontitis [46]. Saliva pH value could be affected by tobacco usage [47,48,49]. According to Kumar et al., saliva of tobacco smoking patients with periodontitis was characterized by a lower pH value than saliva of non-smoking patients with periodontitis [47]. Parvinen et al. [48] and Ömeroğlu et al. [49] proved that saliva pH of traditional cigarette smokers was lower in comparison to non-smokers. However, Nakonieczna et al. did not observe any changes in saliva pH related to traditional cigarette smoking [50]. In our research, no statistically significant differences in saliva pH were observed, however, pH values among traditional cigarette smokers and e-cigarette users were lower than among non-smokers. 

Tobacco smoking also affects the enzymatic abilities of proteins present in saliva, including lactate dehydrogenase, salivary amylase [51,52], and acid phosphatase [41]. Weiner et al. proved that salivary amylase significantly lost its activity as a result of exposure to tobacco smoke [44]. Aldehydes, which are a component of tobacco smoke, are primarily related to the adverse effect on the enzymatic abilities of proteins. They are the main source of double bonds that participate in chemical reactions with the -SH groups of proteins [51,53]. Nakonieczna et al. did not observe any correlation in saliva protein level with traditional cigarettes smoking [50]. However, in research conducted by Kotle et al., a reduced saliva protein level among traditional cigarette smokers with periodontitis compared to non-smokers with periodontitis was found [54]. In our research, total protein concentration in saliva among e-cigarette users was higher than in non-smoker group and lower than in traditional cigarette smokers, therefore, in the group of traditional cigarette smokers, it was higher than among both e-cigarette users and no smokers. However, statistically significant differences were observed only in the group of traditional cigarettes smokers in comparison to non-smokers.

Salivary composition of inorganic components, such as calcium and phosphates, poses an impact on calculus formation, which is a risk factor for both periodontal diseases and dental caries development [55]. The consequence of increasing concentration of calcium and phosphates ions in saliva may accelerate the mineralization of the dental plaque [56,57]. Zuabi et al. observed a reduced level of calcium in the group of traditional cigarette smokers compared to among non-smokers [58]. In research conducted by Kotle et al., a reduced level of calcium in saliva among traditional cigarette smokers with periodontitis compared to non-smokers with periodontitis was found [54]. On the contrary, Shashikanth et al. observed a statistically insignificant increase in the level of calcium among traditional cigarette smokers [56]. Studies conducted by Sevon et al. [59] and McGregor et al. [60] showed a significantly higher concentration of calcium in stimulated saliva among smokers. However, Nakonieczna et al. did not observe any correlation in saliva calcium level with traditional cigarette smoking [50]. In our research, the concentration of calcium in saliva of e-cigarette users was statistically significantly higher than among non-smokers, however, differences between traditional cigarette smokers and non-smokers were not observed. 

Tobacco usage might also affect the concentration of phosphates in saliva. According to Kotle et al., a reduced level of phosphorus in saliva among traditional cigarette smokers with periodontitis compared to non-smokers with periodontitis was found [54]. However, Erdemir et al. [61] did not find any differences in the level of phosphates in saliva between tobacco smokers and non-smokers. In our research, no statistically significant differences in relation to phosphates were observed, however, the concentration of phosphates among e-cigarette users was higher than in groups of traditional cigarette smokers and non-smokers. Phosphate concentration in the group of traditional cigarette smokers was lower than among e-cigarettes users and higher than among non-smokers. 

## 5. Conclusions

Saliva of e-cigarette users present changes in physicochemical composition, including values of pH, and concentration of total protein, calcium, and phosphates, in comparison to traditional cigarette smokers and non-smokers, however, statistically significant differences were observed only in calcium concentration. Further longitudinal studies on a larger group of long-term e-cigarette users should be conducted to assess the effect of the observed results in the physicochemical composition of saliva on oral health, including the potential risk of periodontal diseases development.

## Figures and Tables

**Table 1:** Values of unstimulated saliva pH and concentration of total protein, calcium, and phosphate concentration among e-cigarette users, traditional cigarette smokers, and non-smokers.

		E-Cigarettes Users (n = 40)	Cigarettes Smokers (n = 39)	Non-Smokers (n = 49)	*p*-Value
pH					0.5432 ^1^
	mean (SD)	7.1 (0.7)	7.2 (0.6)	7.3 (0.8)	
	range	5.5–8.0	5.0–8.0	6.0–8.5	
	median	7.0	7.0	7.5	
	95% CI	[6.9;7.3]	[7.0;7.4]	[7.0;7.5]	
Total protein					0.0101 ^1^
	mean (SD)	2.3 (1.2)	2.2 (0.8)	1.7 (0.5)	^a^ 0.0111 ^2^
	range	0.9–7.5	1.1–4.3	1.0–3.0	
	median	1.8	2.1 ^a^	1.7 ^a^	
	95% CI	[1.8;2.7]	[1.9;2.5]	[1.5;1.9]	
Calcium (mM/L)					0.0058 ^1^
	mean (SD)	0.8 (0.5)	0.6 (0.3)	0.6 (0.3)	^b^ 0.0051 ^2^
	range	0.2–3.0	0.2–1.3	0.1–1.6	
	median	0.8 ^b^	0.6	0.6 ^b^	
	95% CI	[0.7;1.0]	[0.5;0.7]	[0.5;0.7]	
Phosphates (mM/L)					0.1187 ^1^
	mean (SD)	4.3 (2.0)	4.1 (2.3)	3.4 (1.5)	
	range	1.1–10.3	1.8–10.5	1.3–7.6	
	median	4.1	3.0	2.9	
	95% CI	[3.7;5.0]	[3.3;4.9]	[3.0;3.9]	

Legend: SD—standard deviation, ^a,b^—testify to statistically significant values; a-a, b-b,—groups with statistical significance, *p* < 0.05 for a-a, *p* < 0.01 for b-b, ^1^—Kruskall–Wallis test, ^2^—Dunn post hoc test.

## Data Availability

Not applicable.
